# Draft genome sequence of the cellulolytic endophyte *Chitinophaga costaii* A37T2^T^

**DOI:** 10.1186/s40793-017-0262-2

**Published:** 2017-09-06

**Authors:** Diogo N. Proença, William B. Whitman, Nicole Shapiro, Tanja Woyke, Nikos C. Kyrpides, Paula V. Morais

**Affiliations:** 1 CEMMPRE, University of Coimbra, 3030-788 Coimbra, Portugal; 2 Department of Microbiology, 527 Biological Sciences Building, University of Georgia, Athens, GA 30602-2605 USA; 3 DOE Joint Genome Institute 2800 Mitchell Drive, Walnut Creek, CA 94598 USA; 4 Department of Life Sciences, FCTUC, Faculty of Sciences and Technology, University of Coimbra, Calçada Martim de Freitas, 3001-401 Coimbra, Portugal

**Keywords:** *Chitinophaga costaii* A37T2, Cellulase, Chitinase, Genome sequence

## Abstract

Here we report the draft genome sequence of *Chitinophaga costai* A37T2^T^ (=CIP 110584^T^, =LMG 27458^T^), which was isolated from the endophytic community of *Pinus pinaster* tree. The total genome size of *C. costaii* A37T2^T^ is 5.07 Mbp, containing 4204 coding sequences. Strain A37T2^T^ encoded multiple genes likely involved in cellulolytic, chitinolytic and lipolytic activities. This genome showed 1145 unique genes assigned into 109 Cluster of Orthologous Groups in comparison with the complete genome of *C. pinensis* DSM 2588^T^. The genomic information suggests the potential of the strain A37T2^T^ to interact with the plant metabolism. As there are only a few bacterial genomes related to Pine Wilt Disease, this work provides a contribution to the field.

## Introduction

The genus 10.1601/nm.8334 belongs to the family *Chtiniphagaceae* (phylum 10.1601/nm.7927) alongside with the genera 10.1601/nm.26356, 10.1601/nm.24507, 10.1601/nm.10262, 10.1601/nm.25037, 10.1601/nm.25347, 10.1601/nm.14773, 10.1601/nm.14303, 10.1601/nm.26907, 10.1601/nm.19904, 10.1601/nm.11311, 10.1601/nm.22146, 10.1601/nm.14308, 10.1601/nm.24623, 10.1601/nm.21260, 10.1601/nm.14305, 10.1601/nm.10412, 10.1601/nm.10256, 10.1601/nm.28007, 10.1601/nm.14937, 10.1601/nm.13147, 10.1601/nm.11309, 10.1601/nm.24621, 10.1601/nm.10067, 10.1601/nm.25337 and *Vibriomonas*. The genus 10.1601/nm.8334 is widely distributed in the environment and strains of this genus have been isolated from pine trees, soil, rhizosphere soil, roots, vermicompost and weathered rock [1]. Twenty-four species belonging to the genus 10.1601/nm.8334 have been described [2], and only the type species of the genus 10.1601/nm.8335 has the complete genome sequenced [3].

*Pinus pinaster* trees from Central Portugal present a diverse endophytic microbial community. Strain A37T2^T^ was isolated as part of the endophytic microbiome of pine trees affected by Pine Wilt Disease (PWD) which is a world devastating disease, consequence of *Bursaphelenchus xylophilus* colonization in pine trees [4]. Here, we show the second genome of the genus 10.1601/nm.8334, a draft genome of 10.1601/nm.25336 A37T2^T^, previously isolated as endophyte of *Pinus pinaster* affected by PWD [1].

## Organism information

### Classification and features

**Fig. 1:**
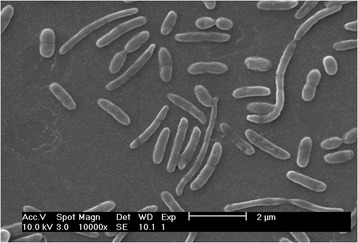
Scanning electron micrograph of *C. costaii* A37T2^T^ after 48 h of growth on R2A agar plates at 30 °C

**Fig. 2:**
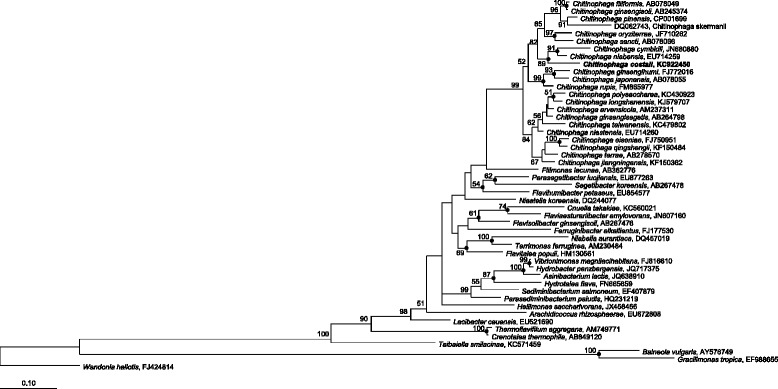
Phylogenetic tree based on a comparison of the 16S rRNA gene sequence of strain A37T2^T^ and the other type strains within the family *Chitinophagaceae*. The tree was created using the maximum likelihood method (RAxML). The numbers on the tree indicate the percentages of bootstrap sampling, derived from 1000 replications; values below 50% are not shown. Symbol (•) indicates node branches conserved when the tree was reconstructed using the neighbor-joining method. The isolate characterized in this study is indicated in bold. Scale bar, 1 inferred nucleotide substitution per 100 nucleotides

The type strain A37T2^T^ (=10.1601/strainfinder?urlappend=%3Fid%3DCIP+110584 ^T^ =10.1601/strainfinder?urlappend=%3Fid%3DLMG+27458 ^T^), was isolated from tree trunk of a *Pinus pinaster* tree affected by PWD and it described as 10.1601/nm.25336 (family 10.1601/nm.14400, phylum 10.1601/nm.7927) [1]. It was Gram-stain-negative, facultative anaerobic, non-motile, formed rod-shaped cells, 0-5-1 μm in diameter and 1-8 μm in length after 48 h on R2A agar media (Fig. 1). Showed capacity to grow on R2A agar medium at 15-45 °C (optimum, 26-30 °C), at pH 5.5-8.0 (optimum, pH 7) and supplemented with up to 1% (*w*/*v*) NaCl (optimum without NaCl). The major fatty acids (>25%) showed by the strain A37T2^T^ are saturated iso-C_15_: _0_ and unsaturated C_16_: _1 *ω5c*_. The major polar lipids were identified as phosphatidylethanolamine, two unidentified aminophospholipids and one unidentified lipid. No glycolipid was detected. The menaquinone 7 (MK-7) was shown as the major respiratory lipoquinone. The determined DNA G + C content of the 10.1601/nm.25336 A37T2^T^ was 46.6 mol%. Key features of this microorganism are summarized in Table 1. A phylogenetic tree based on the 16S rRNA gene sequence of this strain and its closest relative members are given in Fig. 2. The sequences were aligned by SINA (v1.2.9) using the SILVA SEED as reference alignment [5]. Sequences were included in 16S rRNA-based Living Tree Project (LTP) release 115 database [6] by parsimony implemented in the ARB software package version 5.5 [7]. Evolutionary distances were calculated [8] and phylogenetic dendrograms were constructed using the neighbor-joining [9] and Randomized Axelerated Maximum Likelihood (RAxML) method with GTRGAMMA model [10] included in the ARB software [7]. Trees topologies were evaluated by performing bootstrap analysis [11] of 1000 data sets by using ARB software package.

**Table 1:** Classification and general features of *Chitinophaga costaii* A37T2^T^ according to the MIGS recommendations [26]

MIGS ID	Property	Term	Evidence code^a^
	Classification	Domain *Bacteria*	TAS [27]
		Phylum *Bacteroidetes*	TAS [28, 29]
		Class *Sphingobacteriia*	TAS [28, 30]
		Order *Sphingobacteriales*	TAS [28, 31]
		Family *Chitinophagaceae*	TAS [32]
		Genus *Chitinophaga*	TAS [33]
		Species *Chitinophaga costaii*	TAS [1]
		Type strain: A37T2^T^ (=CIP 110584^T^, =LMG 27458^T^)	
	Gram stain	Negative	TAS [1]
	Cell shape	Rod	TAS [1]
	Motility	Non-motile	TAS [1]
	Sporulation	Not reported	NAS
	Temperature range	15-45 °C	TAS [1]
	Optimum temperature	26-30 °C	TAS [1]
	pH range; Optimum	5.5-8.0; 7	TAS [1]
	Carbon source	Glucose	TAS [1]
MIGS-6	Habitat	Endophyte of *Pinus pinaster* tree	TAS [1]
MIGS-6.3	Salinity	1.0% NaCl (*w*/*v*)	TAS [1]
MIGS-22	Oxygen requirement	Facultative anaerobic	TAS [1]
MIGS-15	Biotic relationship	Free-living	TAS [1]
MIGS-14	Pathogenicity	Non-pathogen	NAS
MIGS-4	Geographic location	Portugal	TAS [1]
MIGS-5	Sample collection	July, 2009	NAS
MIGS-4.1	Latitude	40.2962266	NAS
MIGS-4.2	Longitude	−7.9207357	NAS
MIGS-4.4	Altitude	217 m	NAS

^a^Evidence codes - IDA: Inferred from Direct Assay; TAS: Traceable Author Statement (i.e., a direct report exists in the literature); NAS: Non-traceable Author Statement (i.e., not directly observed for the living, isolated sample, but based on a generally accepted property for the species, or anecdotal evidence). These evidence codes are from the Gene Ontology project [34]

## Genome sequencing information

### Genome project history

This Whole Genome Shotgun project has been deposited at ENA under the accession numbers FMAR01000001-FMAR01000056 and in the Integrated Microbial Genomes database (IMG) with Biosample ID SAMN05216457 [12]. The genome sequencing of this organism is part of the Genomic Encyclopedia of Bacteria and Archaea [13], 1000 Microbial Genomes project, phase III (KMG-III) [14], at the U.S. Department of Energy, Joint Genome Institute (JGI). The project information and its association with the MIGS is summarized in Table 2.

**Table 2:** Project information

MIGS ID	Property	Term
MIGS 31	Finishing quality	Draft
MIGS-28	Libraries used	Illumina Regular Fragment, 300 bp, Tubes
MIGS 29	Sequencing platforms	Illumina HiSeq 2500-1 TB
MIGS 31.2	Fold coverage	297.2
MIGS 30	Assemblers	SPAdes
MIGS 32	Gene calling method	NCBI Prokaryotic Genome Annotation Pipeline
	Locus Tag	GA0116948
	Genbank ID	FMAR00000000
	GenBank Date of Release	August 3, 2016
	GOLD ID	Gp0139259
	BIOPROJECT	PRJNA322901
MIGS 13	Source Material Identifier	A37T2^T^
	Project relevance	GEBA-KMG

### Growth conditions and genomic DNA preparation

The strain A37T2^T^ was grown on R2A agar media at 30 °C during 48 h and its genomic DNA was extracted using the E.Z.N.A. Bacterial DNA Kit (Omega Bio-Tek, Norcross, GA, USA) according to the manufacturer’s instructions.

### Genome sequencing and assembly

The draft genome of 10.1601/nm.25336 A37T2^T^ was generated at the DOE Joint Genome Institute (JGI) using the Illumina technology [15]. An Illumina 300 bp insert standard shotgun library was constructed and sequenced using the Illumina HiSeq–2500 1 TB platform, generating 9,965,394 reads totaling 1494.8 Mbp. All general aspects of library construction and sequencing performed at the JGI can be found at [16]. All raw Illumina sequence data was filtered using BBDuk [17], which removes known Illumina artifacts and PhiX. Reads with more than one “N” or with quality scores (before trimming) averaging less than 8 or reads shorter than 51 bp (after trimming) were discarded. Remaining reads were mapped to masked versions of human, cat and dog references using BBMAP [17] and discarded if identity exceeded 95%. Sequence masking was performed with BBMask [17]. Following steps were then performed for assembly: (1) artifact filtered Illumina reads were assembled using SPAdes (version 3.6.2) [18]; (2) assembled contigs were discarded if length was <1 kbp. Parameters for the SPAdes assembly were ––cov–cutoff auto ––phred–offset 33 –t 8 –m 40 ––careful –k 25,55,95 ––12.

### Genome annotation

Protein-coding genes were identified using Prodigal [19], as part of the DOE-JGI genome annotation pipeline [20]. Additional gene prediction analysis and manual functional annotation were performed within the Integrated Microbial Genomes Expert Review system (IMG-ER), which provides tools for analyzing and reviewing the structural and functional annotations of genomes in a comparative context [12, 21]. Genome annotation procedures are detailed in Markowitz et al. [12] and references therein. Briefly, the predicted CDSs were translated and used to search the NCBI nonredundant database, UNIProt, TIGRFam, Pfam, KEGG, COG and InterPro databases. Transfer RNA genes were identified using the tRNAScan-SE tool and other non-coding RNAs were found using INFERNAL. Ribosomal RNA genes were predicted using hmmsearch against the custom models generated for each type of rRNA.

### Genome properties

The draft genome sequence of 10.1601/nm.25336 strain A37T2^T^ comprised 5,074,440 bp, based on 1494.8 Mbp of Illumina data with a mapped coverage of 297.2-fold of the genome. The final draft assembly contained 56 contigs in 56 scaffolds with more than 1052 bp. The G + C content was 47.6%. The genome encoded 4204 putative coding sequences (CDSs) (Table 3). Fifty four % of the CDSs, corresponding to 2284 proteins, could be assigned to Cluster of Orthologous Groups (COG) families [22] (Table 4). The draft genome sequence contained four ribosomal RNAs and 50 tRNAs loci (Table 3).

**Table 3:** General genome features of *Chitinophaga costaii* A37T2^T^

Attribute	Value	% of Total
Genome size (bp)	5,074,440	100.00
DNA coding (bp)	4,431,743	87.33
DNA G + C (bp)	2,413,598	47.56
DNA scaffolds	56	100.00
Total genes	4274	100.00
Protein coding genes	4204	98.36
RNA genes	70	1.64
Genes in internal clusters	824	19.28
Genes with function prediction	3041	71.15
Genes assigned to COGs	2284	53.44
Genes with Pfam domains	1976	61.75
Genes with signal peptides	651	15.23
Genes with transmembrane helices	972	22.74
CRISPR repeats	3	0.00

**Table 4:** Number of genes associated with general COG functional categories

Code	Value	%age	Description
J	186	7.42	Translation, ribosomal structure and biogenesis
A	0	0.00	RNA processing and modification
K	215	8.58	Transcription
L	90	3.50	Replication, recombination and repair
D	19	0.76	Cell cycle control, Cell division, chromosome partitioning
V	98	3.91	Defense mechanisms
T	114	4.55	Signal transduction mechanisms
M	211	8.42	Cell wall/membrane biogenesis
N	12	0.48	Cell motility
U	20	0.80	Intracellular trafficking and secretion
O	136	5.42	Posttranslational modification, protein turnover, chaperones
C	126	5.03	Energy production and conversion
G	165	6.58	Carbohydrate transport and metabolism
E	196	7.82	Amino acid transport and metabolism
F	69	2.75	Nucleotide transport and metabolism
H	141	5.62	Coenzyme transport and metabolism
I	125	4.99	Lipid transport and metabolism
P	150	5.98	Inorganic ion transport and metabolism
Q	70	2.79	Secondary metabolites biosynthesis, transport and catabolism
R	248	9.89	General function prediction only
S	104	4.15	Function unknown
-	1990	46.56	Not in COGs

The total is based on the total number of protein coding genes in the genome

**Fig. 3:**
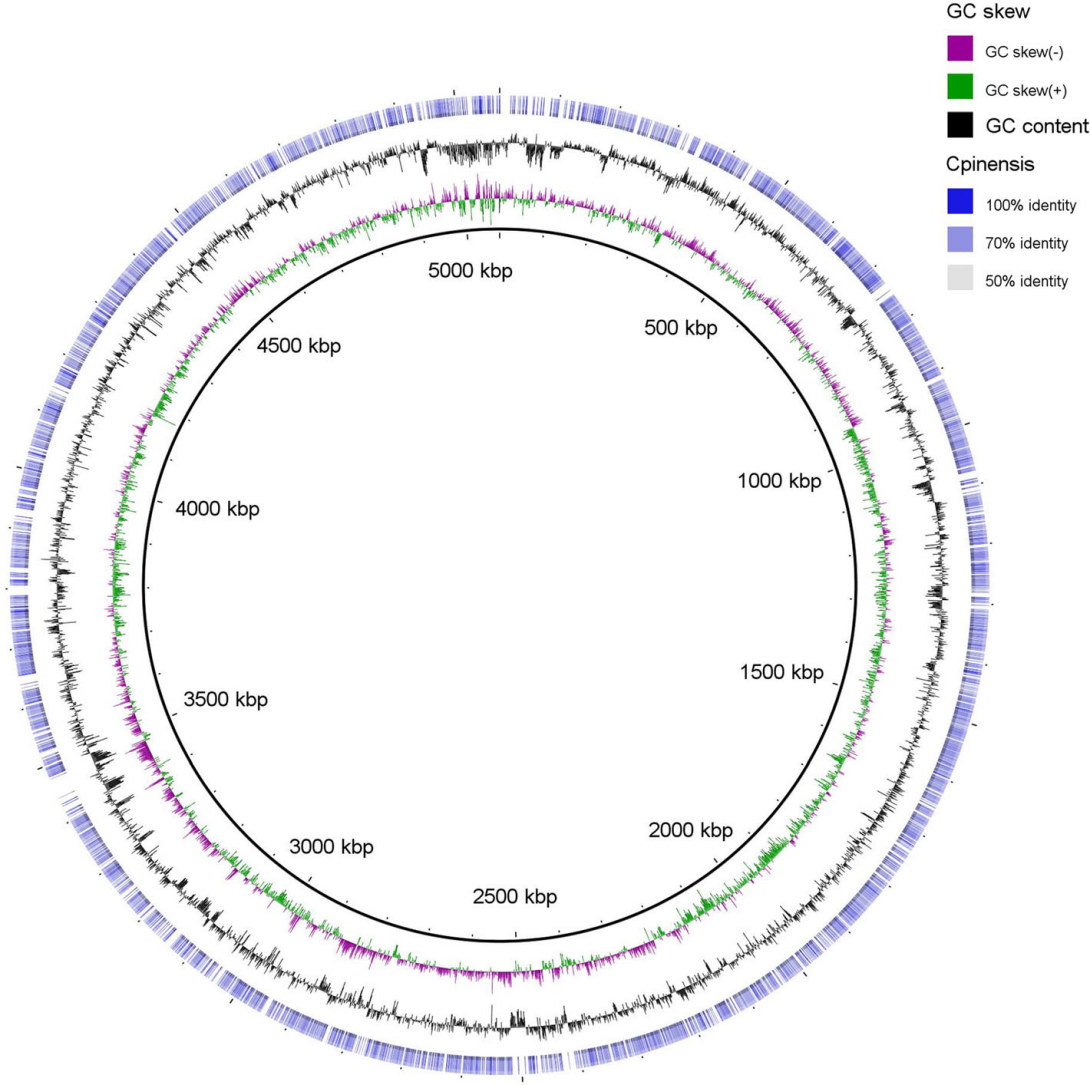
The genome of *Chitinophaga costaii* A37T2^T^. From outside to the center: genes of genome of *C. pinensis* DSM 2588^T^ and its similarity with the genome of *C. costaii* (50-100%), GC content of *C. costaii* A37T2^T^, GC skew of *C. costaii* A37T2^T^, genome of *C. costaii* A37T2^T^

The Average Nucleotide Identity between 10.1601/nm.25336 A37T2^T^ and 10.1601/nm.8335 10.1601/strainfinder?urlappend=%3Fid%3DDSM+2588 ^T^ was 70.9 based on 1593 of total Bidirectional Best Hits, using MiSI [23]. Figure 3 shows the circular graph of the genome of 10.1601/nm.25336 A37T2^T^ query to the only available complete genome of the genus 10.1601/nm.8334, 10.1601/nm.8335 10.1601/strainfinder?urlappend=%3Fid%3DDSM+2588 ^T^ [2].

The comparison between the draft genome of 10.1601/nm.25336 A37T2^T^ and the complete genome of 10.1601/nm.8335 10.1601/strainfinder?urlappend=%3Fid%3DDSM+2588 ^T^ showed 1145 unique genes only present in the genome of 10.1601/nm.25336 A37T2^T^ and 3493 unique genes only present in the genome of 10.1601/nm.8335 10.1601/strainfinder?urlappend=%3Fid%3DDSM+2588 ^T^. Focused on the unique genes present on the genome of strain A37T2^T^ it was possible to assigned 109 COG, summarized in Table 5.

**Table 5:** Unique Cluster Orthologous Groups present in the genome of *C. costaii* A37T2^T^

Catergory Code	Catergory	COG ID
C	Energy production and conversion	COG0280, COG0374, COG0680, COG1740
E	Amino acid transport and metabolism	COG1027, COG1586, COG2355, COG3104
F	Nucleotide transport and metabolism	COG0027
G	Carbohydrate transport and metabolism	COG0021, COG0058, COG0588, COG0662, COG0837, COG1080, COG1803, COG1925, COG2079, COG2893, COG3444, COG3716, COG3934
H	Coenzyme transport and metabolism	COG0561, COG1056, COG2091, COG2227, COG2329
I	Lipid transport and metabolism	COG0671, COG0821, COG2246
J	Translation, ribosomal structure and biogenesis	COG0060, COG0255, COG0257, COG0267, COG0268, COG0333, COG4680
K	Transcription	COG1476, COG4933
L	Replication, recombination and repair	COG0863, COG1722
M	Cell wall/membrane/envelope biogenesis	COG1083, COG1922, COG2089, COG2829, COG2982, COG3511, COG3637
O	Posttranslational modification, protein turnover, chaperones	COG0068, COG0298, COG0309, COG0409
P	Inorganic ion transport and metabolism	COG0428, COG1218, COG1230, COG1416, COG4772
Q	Secondary metabolites biosynthesis, transport and catabolism	COG2130, COG2162, COG3733, COG4242
R	General function prediction only	COG0312, COG0375, COG0429, COG0457, COG1062, COG1373, COG2320, COG3153, COG3488, COG4674, COG0561, COG2130, COG4242
S	Function unknown	COG0393, COG1286, COG2442, COG2962, COG3219, COG3247, COG3310, COG3361, COG3461, COG3477, COG3487, COG3489, COG3528, COG3548, COG3918, COG3943, COG4487, COG4700, COG4859, COG4924
T	Signal transduction mechanisms	COG0517, COG2184, COG2203, COG3292, COG1925
U	Intracellular trafficking, secretion, and vesicular transport	COG1272, COG1826, COG3451
V	Defense mechanisms	COG0286, COG0610, COG0732, COG3512, COG3513, COG4823, COG5499
X	Mobilome: prophages, transposons	COG3385, COG3436, COG3600, COG3654

## Insights from the genome sequence

The draft genome sequence of 10.1601/nm.25336 A37T2^T^ carries multiple genes involved in cellulolytic activity, including one gene encoding the enzyme cellulase (SCC15587) and six genes encoding for β-glucosidase (SCB82491, SCB92249, SCB95191, SCC15475, SCC57293, SCC61957), which might be involved in cellulose degradation in the environment and in biotechnological processes [24]. As expected for this genus, four genes encoding chitinases (SCC19468, SCC19522, SCC23114, SCC34676) were found. Six genes encoded lysophospholipase L1, including representatives of both of size groups, i.e. less than 300aa (SCB77875, SCC28514, SCC37316, SCC54197) and less than 500aa (SCB98645, SCC50813). Moreover, the genome of strain A37T2^T^ encoded 1-aminocyclopropane-1-carboxylate deaminase (SCB80758), a hydrolase that might be involved in lowering ethylene levels in the plant [25]. In summary, the genome sequence suggested multiple potentials for the strain to interact with the plant metabolism.

## Conclusions

This work contributed to the knowledge of the genome sequence of the type species of 10.1601/nm.25336 A37T2^T^ (=10.1601/strainfinder?urlappend=%3Fid%3DCIP+110584 ^T^, =10.1601/strainfinder?urlappend=%3Fid%3DLMG+27458 ^T^), an endophyte of *P. pinaster* affected by PWD. The genome encoded multiple genes involved in cellulolytic activity and the sequence provided insights into the role of bacteria in PWD. As there are only a few bacterial genomes related to PWD, this work provides a contribution to this field.

## Abbreviations

PWD: Pine wilt disease
PWN: Pinewood nematode
